# Pseudomonas aeruginosa Quorum Sensing Molecule Alters Skeletal Muscle Protein Homeostasis by Perturbing the Antioxidant Defense System

**DOI:** 10.1128/mBio.02211-19

**Published:** 2019-10-01

**Authors:** Arunava Bandyopadhaya, A. Aria Tzika, Laurence G. Rahme

**Affiliations:** a Department of Surgery, Center for Surgery, Innovation and Bioengineering, Massachusetts General Hospital, Harvard Medical School Boston, Massachusetts, USA; b Shriners Hospitals for Children Boston, Boston, Massachusetts, USA; c Athinoula A. Martinos Center of Biomedical Imaging, Massachusetts General Hospital, Boston, Massachusetts, USA; d Department of Microbiology, Harvard Medical School, Boston, Massachusetts, USA; Harvard Medical School; University of Manitoba; University of Chicago

**Keywords:** 2-amino acetophenone (2-AA), NAD(P)H oxidase 2 (NOX2), *Pseudomonas aeruginosa*, skeletal muscle, xanthine oxidase (XO), chronic infections, muscle atrophy, muscle dysfunction, quorum sensing (QS), reactive oxygen species (ROS), virulence

## Abstract

Skeletal muscle function is compromised in many illnesses, including chronic infections. The Pseudomonas aeruginosa quorum sensing (QS) signal, 2-amino acetophenone (2-AA), is produced during acute and chronic infections and excreted in human tissues, including the lungs of cystic fibrosis patients. We have shown that 2-AA facilitates pathogen persistence, likely via its ability to promote the formation of bacterial persister cells, and that it acts as an interkingdom immunomodulatory signal that epigenetically reprograms innate immune functions. Moreover, 2-AA compromises muscle contractility and impacts the expression of genes involved in reactive oxygen species (ROS) homeostasis in skeletal muscle and in mitochondrial functions. Here, we elucidate the molecular mechanisms of 2-AA’s impairment of skeletal muscle function and ROS homeostasis. Murine *in vivo* and differentiated C2C12 myotube cell studies showed that 2-AA promotes ROS generation in skeletal muscle via the modulation of xanthine oxidase (XO) activity, NAD(P)H oxidase2 (NOX2) protein level, and the activity of antioxidant enzymes. ROS accumulation triggers the activity of AMP-activated protein kinase (AMPK), likely upstream of the observed locations of induction of ubiquitin ligases Muscle RING Finger 1 (MuRF1) and Muscle Atrophy F-box (MAFbx), and induces autophagy-related proteins. The protein-level perturbation in skeletal muscle of silent mating type information regulation 2 homolog 1 (SIRT1), peroxisome proliferator-activated receptor gamma coactivator 1 (PGC-1), and uncoupling protein 3 (UCP3) is rescued by the antioxidant N-acetyl-l-cysteine (NAC). Together, these results unveil a novel form of action of a QS bacterial molecule and provide molecular insights into the 2-AA-mediated skeletal muscle dysfunction caused by P. aeruginosa.

## Abstract (precis)

Pseudomonas aeruginosa, a bacterium that is resistant to treatment, causes serious acute, persistent, and relapsing infections in humans. There is increasing evidence that bacterial excreted small molecules play a critical role during infection. We have shown that a quorum sensing (QS)-regulated excreted small molecule, 2-AA, which is abundantly produced by P. aeruginosa, promotes persistent infections, dampens host inflammation, and triggers mitochondrial dysfunction in skeletal muscle. QS is a cell-to-cell communication system utilized by bacteria to promote collective behaviors. The significance of our study in identifying a mechanism that leads to skeletal muscle dysfunction, via the action of a QS molecule, is that it may open new avenues in the control of muscle loss as a result of infection and sepsis. Given that QS is a common characteristic of prokaryotes, it is possible that 2-AA-like molecules promoting similar effects may exist in other pathogens.

## INTRODUCTION

Skeletal muscle function, which is essential for survival, is compromised in both acute and chronic infections (1–4). Bacteria, as well as bacterial cell membrane constituents such as lipopolysaccharide (LPS), disrupt mitochondrial physiology in skeletal muscle, resulting in mitochondrial dysfunction and potentially in muscle atrophy (5–7). Severe sepsis results in muscle mass loss and later muscle wasting due to increased proteolysis (8, 9). Sepsis survivors exhibit severe muscle weakness that overall remains poorly understood.

Reactive oxygen species (ROS), which are generated at multiple subcellular locations in skeletal muscle, have been associated with both physiological functions and pathology of this tissue (10, 11). The first biological redox system involved, in the context of defense time line, is comprised of O_2_^-^, H_2_O_2_, and other reactive oxygen radicals, collectively known as ROS. During homeostasis, an overall oxidative balance is maintained in the tissue by limiting the production of ROS via a variety of antioxidant defense systems. However, when ROS levels are increased, they activate two major protein degradation pathways, the ubiquitin-proteasome pathway and the autophagy-lysosome pathway, while they impair mitochondrial functions that are implicated in muscle mass loss and atrophy (12, 13). Both the ubiquitin-proteasome pathway, which is mostly responsible for myofilament protein degradation, and the autophagy pathway, which is a critical regulator of protein homeostasis and mitochondrial quality control, are also promoted by bacterial infections (14–17).

2-Amino acetophenone (2-AA) is a quorum sensing (QS)-regulated low-molecular-weight bacterial molecule (18), produced and excreted by Pseudomonas aeruginosa, a highly problematic pathogen that defies efforts at antibiotic-based eradication. QS is a bacterial cell-to-cell communication system governed by population density that regulates many virulence factors in synchrony (19–24). We have shown previously that 2-AA negatively impacts the expression of both muscle contraction-related genes and muscle development-related genes along with the genes involved in energy production, oxidative phosphorylation, ROS homeostasis, and intermediate metabolism in skeletal muscle (25, 26). *In vivo* nuclear magnetic resonance (NMR) studies have shown that 2-AA reduced the ATP synthesis rate in skeletal muscle, while functional muscle studies have suggested that this bacterially excreted small molecule can compromise muscle contractility. These results, along with the gene expression findings, further indicate that 2-AA impairs skeletal muscle activity via mitochondrion-related functions (25, 26).

P. aeruginosa promotes acute and chronic infections in immunocompromised patients, including those suffering from cystic fibrosis (CF). CF patients are frequently and chronically infected with multidrug-resistant P. aeruginosa strains and suffer from significant skeletal muscle wasting in the later stage of the disease (27, 28). Interestingly, 2-AA enables the bacteria to persist over the long term in infected tissues through a distinct molecular mechanism of host chromatin regulation (18, 29, 30). The mitochondrial dysfunction promoted by the P. aeruginosa molecule 2-AA may further favor infection and may represent an important step in the establishment of chronic/persistent infections (25, 26, 29, 30).

Here, we interrogate the components involved in and the mechanistic aspects that contribute to ROS accumulation and oxidative stress in response to this bacterial QS molecule that we found to compromise skeletal muscle functions (25). Understanding the role of this molecule in the promotion of muscle dysfunction may open new avenues in the control of muscle loss as a result of infection and sepsis.

## RESULTS

### 2-AA triggers ROS overproduction in murine skeletal muscle, which can be counteracted by antioxidant treatment.

**FIG 1:**
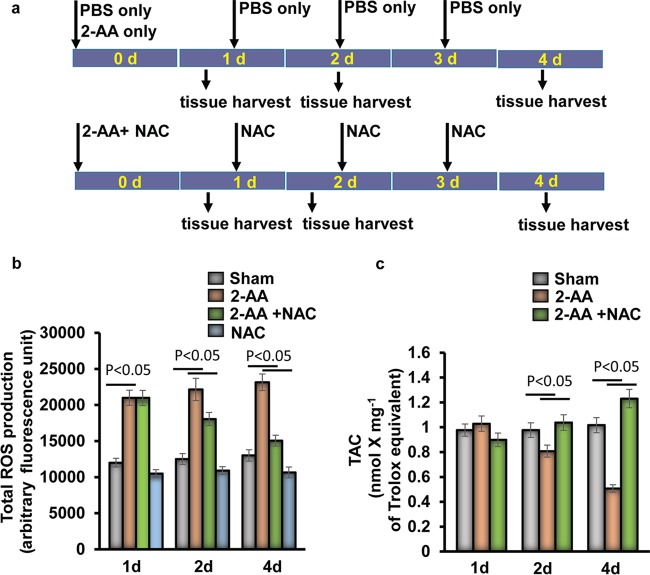
Antioxidant treatment rescues 2-AA-induced oxidative stress in murine gastrocnemius muscle. (a) Schematic diagram of 2-AA and NAC treatment in mice. Mice were injected with 2-AA (6.75 mg/kg; i.p.) once, and NAC was given (10 mg/kg; i.p.) once per day. (b and c) ROS production (b) and TAC (c) were measured in skeletal muscle 1 day (1d), 2 days (2d), and 4 days (4d) after 2-AA treatment and 2-AA-plus-NAC treatment (*n* = 5). The results were expressed as means ± standard deviations (SD). *P* < 0.05, one-way ANOVA.

We measured ROS production in gastrocnemius muscle following the experimental design presented in Fig. 1a. In agreement with the prior finding that *PGC-1* gene downregulation led to decreased expression of genes involved in detoxification of ROS and toxic oxidizing species (25, 26), we observed that 2-AA increased the levels of ROS production in gastrocnemius muscle tissues measured at 1 day, 2 days, and 4 days after 2-AA treatment and that ROS production levels were sustained during the course of the 4 days that were assessed (Fig. 1b). Antioxidant N-acetyl cysteine (NAC) treatment diminished ROS production in 2-AA-injected mice (Fig. 1b). To determine the capacity of the endogenous antioxidant systems to counterbalance the deleterious effects of oxidative stress in skeletal muscle due to induction of ROS following 2-AA injection, we assessed the total antioxidant status in skeletal muscle of 2-AA-treated and untreated mice by using the total antioxidant capacity (TAC) assay. There were no significant differences in the levels of total antioxidant capacity observed in comparisons of control animals to 2-AA-treated animals at 1 day or 2 days (Fig. 1c). However, as shown in Fig. 1c, the total antioxidant capacity was significantly compromised in the gastrocnemius 4 days after 2-AA treatment, while NAC treatment prevented the 2-AA-mediated reduction in antioxidant capacity in gastrocnemius muscle at 4 days after 2-AA treatment. This set of data further supports the notion that 2-AA-meditated ROS production promotes oxidative stress in skeletal muscle.

### 2-AA impacts the activity of detoxifying enzymes in skeletal muscle.

**FIG 2:**
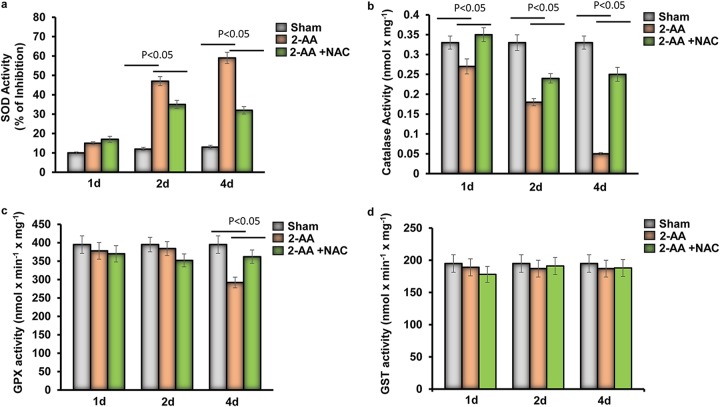
Antioxidant enzyme activity in skeletal muscle of 2-AA-treated mice. (a) Levels of SOD (percent inhibition), (b) catalase, (c) GPX, and (d) GST activity were measured in skeletal muscle 1 day, 2 days, and 4 days after 2-AA treatment and 2-AA-plus-NAC treatment (*n* = 5). The results were expressed as means ± SD. *P* < 0.05, one-way ANOVA.

Superoxide dismutase (SOD), catalase (CAT), glutathione peroxidase (GPX), and glutathione *S*-transferase (GST) represent a collection of antioxidant enzymes that regulate the homeostatic balances between free radicals or reactive species and antioxidants (10). SOD activity was significantly inhibited in 2-AA-treated skeletal muscle at 2 days and 4 days, while NAC partially rescued the SOD activity (Fig. 2a). In addition, catalase activity was significantly and gradually downregulated over the course of 4 days after 2-AA treatment in skeletal muscle and was found to have reached a minimal level by day 4, whereas NAC treatment rescued the catalase activity after 2-AA treatment in the gastrocnemius muscle of mice (Fig. 2b). While the GPX activity was reduced significantly in skeletal muscle 4 days after 2-AA treatment (Fig. 2c), no significant difference was found in GST in skeletal muscle 1 day, 2 days, and 4 days after 2-AA treatment (Fig. 2d). This set of data strongly supports the notion that accumulation of ROS in the skeletal muscle of 2-AA-treated mice is due to impaired activity of SOD, catalase, and GPX enzymes.

### 2-AA impacts xanthine oxidase (XO) activity and NAD(P)H oxidase protein expression in murine skeletal muscle.

**FIG 3:**
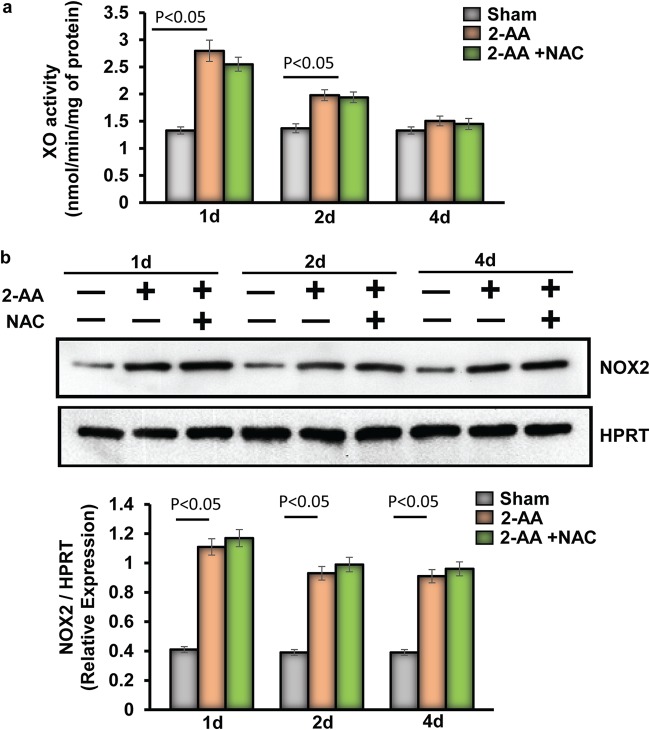
Assessment of XO activity and NOX2 protein expression in skeletal muscle of 2-AA-treated mice. (a) XO activity was measured in skeletal muscle 1 day, 2 days, and 4 days after 2-AA treatment and 2-AA-plus-NAC treatment (*n* = 5 in each group). The results were expressed as means ± SD. *P* < 0.05, one-way ANOVA. (b) Representative immunoblot of NOX2 in skeletal muscle 1 day, 2 days, and 4 days after 2-AA treatment and 2-AA-plus-NAC treatment. HPRT (bottom) was used as the loading control. Histograms show the relative expression levels of proteins. *n* = 5 in each group; data represent means ± SD. *P* < 0.05, one-way ANOVA.

XO and multimeric NOX catalyze the conversion of O_2_ to superoxide and increase oxidative stress levels (31). Therefore, using 2-AA-treated mice, we assessed in skeletal muscle the activity of XO, a cellular enzyme that generates ROS. Panel a of Fig. 3 shows that the XO activity was highly upregulated at 1 day and was found to have gradually declined at 2 days and 4 days after 2-AA treatment, providing a possible explanation for the early production of ROS and the oxidative stress observed in skeletal muscle. However, NAC treatment was not effective in restoring the XO activity (Fig. 3a), suggesting that the activity of this enzyme is not triggered by the ROS production but rather by 2-AA itself.

NAD(P)H oxidase (NOX)2 acts as the main source of skeletal muscle ROS production (32). NOX2 protein levels were increased in skeletal muscle at 1 day and 2 days after 2-AA treatment. Notably, however, NAC treatment did not affect NOX2 protein expression induced by 2-AA (Fig. 3b), demonstrating that 2-AA modulates the expression of NOX2 to induce ROS production in skeletal muscle.

### XO and NOX2 induce 2-AA-mediated ROS levels in murine C2C12 myotubes.

**FIG 4:**
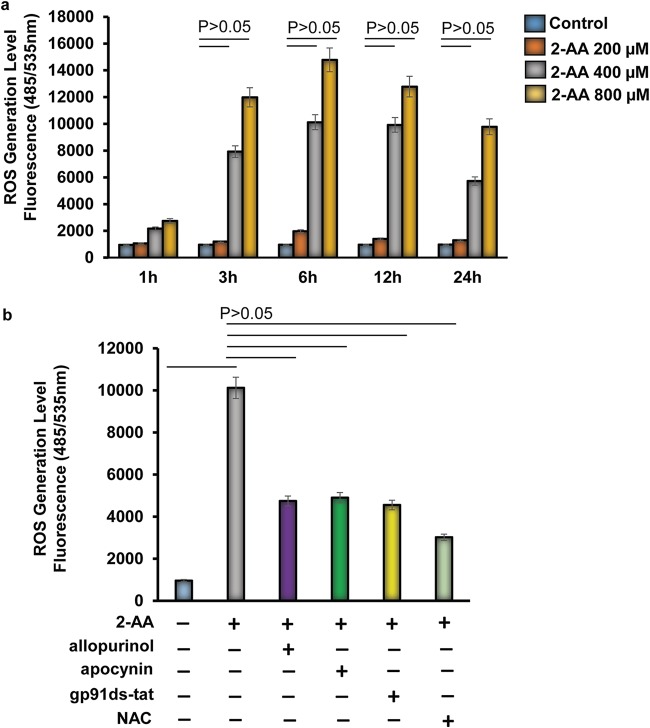
XO and NOX2 inhibitors support the involvement of XO and NOX2 in the 2-AA-mediated initial cellular ROS production in skeletal muscle. (a) Cellular ROS production was measured in differentiated C2C12 myotubes 1 h, 2 h, 3 h, 6 h, 12 h, and 24 h after 2-AA treatment (*n* = 3; values represent means ± SD; *P < *0.05, Student's *t* test). (b) Inhibition of cellular ROS generation was measured in 2-AA (400 μM)-treated cells with or without allopurinol (50 μM), with or without apocynin (10 μM), with or without gp91ds-tat (50 μM), and with or without NAC (5 mM) for 6 h (*n* = 3; values represent means ± SD; *P < *0.05, one-way ANOVA).

Modulation of XO activity and NOX2 protein expression in 2-AA-treated skeletal muscle prompted us to analyze the role of XO and NOX2 in 2-AA-mediated ROS generation. We assessed the time-dependent and dose-dependent effects of 2-AA-mediated ROS generation on murine differentiated C2C12 myotubes (Fig. 4; see also Fig. S1 in the supplemental material). Induction of ROS generation was observed at different concentrations of 2-AA, but significant ROS generation was observed only with 2-AA stimulation at 400 μM and 800 μM (Fig. 4a). ROS generation peaked between 3 and 6 h, with a subsequent gradual decline (Fig. 4a).

10.1128/mBio.02211-19.1 FIG S1 Images of undifferentiated and differentiated mouse C2C12 myoblasts. Bright-field images of a monolayer of undifferentiated C2C12 myoblasts (a) and monolayer of differentiated C2C12 myotubes (b) are shown. Download FIG S1, TIF file, 2.1 MB. Copyright © 2019 Bandyopadhaya et al. 2019 Bandyopadhaya et al. This content is distributed under the terms of the Creative Commons Attribution 4.0 International license.

To identify the molecular pathways for 2-AA-mediated initial ROS generation, we used allopurinol, a specific XO inhibitor (33); apocynin, a nonspecific inhibitor of NOX (34); and gp91ds-tat, a specific NOX2 inhibitor (35). NAC (ROS scavenger) was used to scavenge the ROS as a control (Fig. 4b; see also Fig. S2). As shown in Fig. 4b, 2-AA-mediated ROS generation was blocked by allopurinol, gp91ds-tat, and apocynin, suggesting specific involvement of XO and NOX2 in initial cellular ROS production in 2-AA-treated skeletal muscle cells. Modulation of ROS levels following treatment with 2-AA and inhibitors was not due to cytotoxicity as measured by MTT [3-(4,5-dimethyl-2-thiazolyl)-2,5-diphenyl-2H-tetrazolium bromide] assay (Fig. S3).

10.1128/mBio.02211-19.2 FIG S2 NAC treatment reduces cellular ROS levels in C2C12 myotubes. Pyocyanin (250 μM), a Pseudomonas aeruginosa-excreted phenazine, was used to trigger generation of ROS and to test the activity of NAC. ROS levels triggered by pyocyanin were significantly lower in cells treated with NAC 5 mM. Data represent means ± SD of results from three independent experiments. Download FIG S2, TIF file, 2.3 MB. Copyright © 2019 Bandyopadhaya et al. 2019 Bandyopadhaya et al. This content is distributed under the terms of the Creative Commons Attribution 4.0 International license.

10.1128/mBio.02211-19.3 FIG S3 Assessment of 2-AA and inhibitors of cell viability. An MTT assay measuring viability in differentiated C2C12 myotubes cells after treatment with (a) 2-AA and (b) inhibitors for 48 h was performed. Data represent means ± SD of results from three independent experiments. Download FIG S3, TIF file, 2.1 MB. Copyright © 2019 Bandyopadhaya et al. 2019 Bandyopadhaya et al. This content is distributed under the terms of the Creative Commons Attribution 4.0 International license.

### 2-AA treatment modulates regulators of oxidative metabolism in murine skeletal muscle.

**FIG 5:**
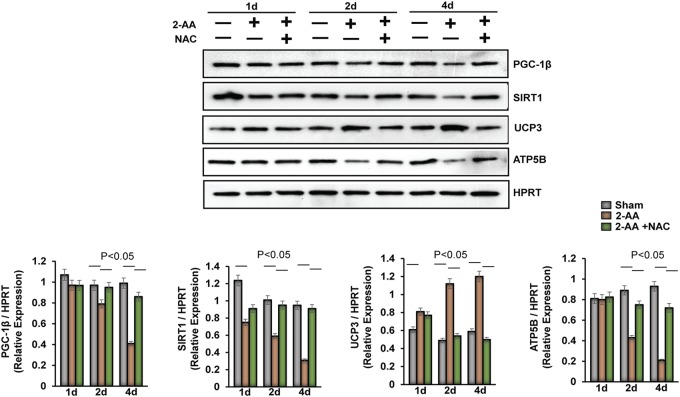
Assessment of the expression of oxidative metabolic regulators in skeletal muscle of 2-AA-treated mice. (Top) Representative immunoblots of PGC-1β, UCP3, SIRT1, and ATP5B in skeletal muscle 1 day, 2 days, and 4 days after 2-AA treatment and 2-AA-plus-NAC treatment. HPRT (bottom blot) was used as the loading control. (Bottom) Histograms show the relative expression levels of proteins. *n* = 5 in each group; data represent means ± SD. *P* < 0.05, one-way ANOVA.

We have previously reported that 2-AA modulates the gene expression of key regulators of oxidative metabolism (PGC-1β, UCP3, and SIRT1) in skeletal murine muscle (25). Here, we assessed their protein levels following NAC treatment (Fig. 5). The dampening of PGC-1β protein levels observed at 2 days and 4 days after 2-AA treatment was partially rescued by NAC (Fig. 5). In addition, the baseline expression of SIRT1 was found to have decreased after 2-AA treatment, and NAC restored SIRT1 levels at 2 days and 4 days (Fig. 5). In contrast, the level of the UCP3 protein, which may participate in a counterregulatory mechanism that functions to lower the production of ROS (36), was further reduced by NAC treatment at 2 days and 4 days after 2-AA treatment (Fig. 5). Finally, NAC treatment also partially restored the level of ATP synthase subunit beta (ATP5b), which is related to oxidative metabolism, at 2 days and 4 days after 2-AA treatment (Fig. 5). These results corroborate our previous findings and strongly support the concept that 2-AA impacts the metabolic regulatory circuit of oxidative metabolism and affects the tissue oxidative status.

### 2-AA treatment induces activation of AMPK in murine skeletal muscle.

**FIG 6:**
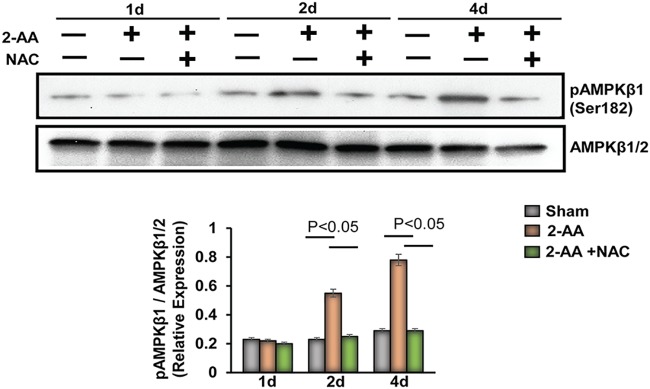
Activation of AMPK in skeletal muscle following 2-AA treatment. (Top) Representative immunoblots of pAMPKβ1 (Ser182) in skeletal muscle 1 day, 2 days, and 4 days after 2-AA treatment and 2-AA-plus-NAC treatment. AMPKβ1/2 was used as a control. (Bottom) Histograms show the relative expression levels of proteins. *n* = 5 in each group; data represent means ± SD. *P* < 0.05, one-way ANOVA.

2-AA treatment stimulates AMP-activated protein kinase (AMPK), which is triggered by reduced ATP levels and oxidative stress, and plays a critical role in cell growth, metabolism reprogramming, autophagy, and maintenance of muscle mass (37, 38). Levels of phosphorylation of AMPKβ1 (Ser182) in the gastrocnemius of mice 2 days and 4 days after 2-AA treatment are shown in Fig. 6. Notably, NAC administration in the 2-AA-treated mice dampened activation of pAMPKβ1, indicating that ROS accumulation could activate the AMPK pathway following 2-AA treatment.

### 2-AA treatment dampens contractile muscle protein expression, while antioxidant treatment provides partial rescue.

**FIG 7:**
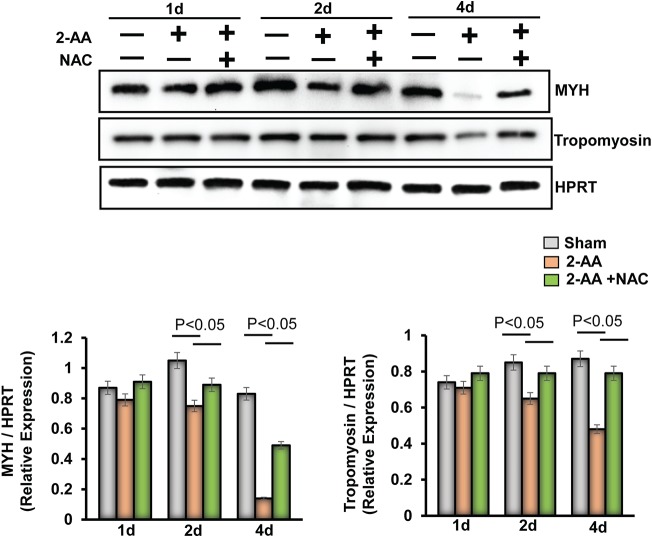
Antioxidant treatment reduces the degradation of muscle protein in gastrocnemius muscle promoted by 2-AA. (Top) Immunoblots of MYH and tropomyosin in skeletal muscle 1 day, 2 days, and 4 days after 2-AA treatment and 2-AA-plus-NAC treatment. HPRT (bottom blot) was used as the loading control. (Bottom) Histograms show the relative expression levels of proteins. *n* = 5 in each group; data represent means ± SD. *P* < 0.05, one-way ANOVA.

We have previously shown that the metabolic effects of 2-AA impact muscle function directly (25) and that the levels of expression of both muscle contraction-related and muscle development-related genes were downregulated 4 days after 2-AA treatment. Immunoblots of myosin heavy chain (MYH) and tropomyosin proteins in gastrocnemius murine muscle 1 day, 2 days, and 4 days after 2-AA treatment demonstrate that 2-AA decreases the expression of crucial contractile proteins at 4 days (Fig. 7), in agreement with our previously published transcriptomic and muscle function data (25). NAC treatment resulted in a partial recovery of the expression of these crucial contractile proteins (Fig. 7).

### 2-AA promotes muscle protein degradation.

**FIG 8:**
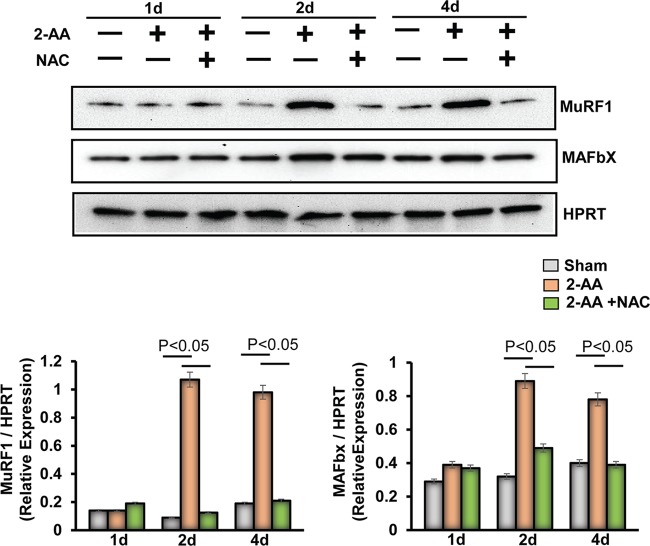
Antioxidant treatment reduces the activation of a ubiquitin-mediated proteasomal pathway in gastrocnemius muscle promoted by 2-AA. (Top) Immunoblot of MuRF1 and MAFbx in skeletal muscle 1 day, 2 days, and 4 days after 2-AA treatment and 2-AA-plus-NAC treatment. HPRT (bottom blot) was used as the loading control. (Bottom) Histograms show the relative expression levels of proteins. *n* = 5 in each group; data represent means ± SD. *P* < 0.05, one-way ANOVA.

2-AA compromises muscle contractility, which in turn may jeopardize muscle functions (25) in a fashion similar to that seen in atrophy (i.e., in the setting of metabolic acidosis, denervation, injury, disuse, hyperthyroidism, excess glucocorticoids, diabetes, cancer, aging, burns, and sepsis (39). This fact prompted us to investigate ubiquitin ligases, which have been shown to mediate the proteasome-dependent protein degradation in muscle wasting (40), as ubiquitin-proteasome and autophagy-lysosome systems are coordinately activated in atrophying muscles (41). 2-AA treatment induced expression of Muscle RING Finger 1 (MuRF1) and of Muscle Atrophy F-box (MAFbx) at 2 days and 4 days following treatment (Fig. 8). This induction corroborates the aforementioned concomitant decrease in MYH levels (Fig. 7), suggesting that at least MuRF1 may contribute to loss of MYH protein, which physically associates with MuRF1. NAC treatment was found to have dampened the 2-AA-induced levels of MuRF1 and MAFbx protein expression at 2 days and 4 days (Fig. 8), suggesting that 2-AA-mediated ROS may reduce contractile muscle protein expression via inducing ubiquitin ligases in skeletal muscle.

**FIG 9:**
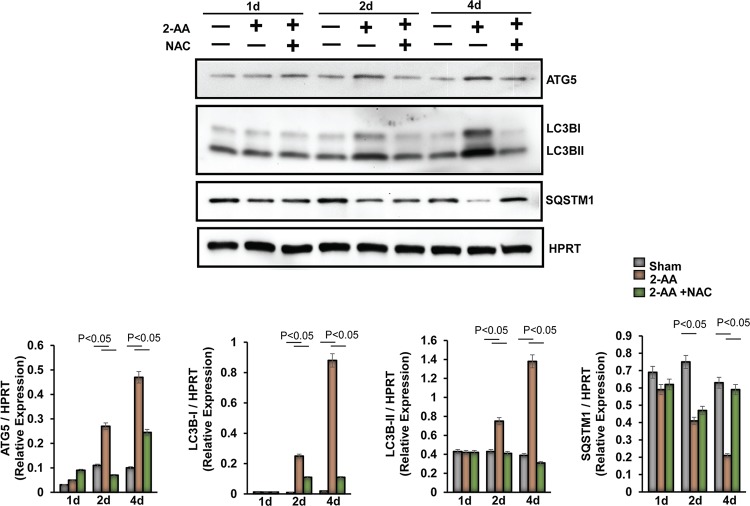
Activation of autophagy markers in skeletal muscle following 2-AA and NAC treatment. (Top) Representative immunoblots of ATG5, LC3B, and SQSTM1/p62 in skeletal muscle 1 day, 2 days, and 4 days after 2-AA treatment and 2-AA-plus-NAC treatment. HPRT (bottom blot) was used as the loading control. (Bottom) Histograms show the relative expression levels of proteins. *n* = 5 in each group; data represent means ± SD. *P* < 0.05, one-way ANOVA.

We next assessed the impact of 2-AA on protein degradation and autophagy in murine skeletal muscle (Fig. 9). First, we assessed the changes in expression levels of autophagic markers in the 2-AA-treated muscle. Autophagy-related protein 5 (ATG5) levels increased 2 days after 2-AA treatment, whereas NAC treatment dampened ATG5 protein expression (Fig. 9). To measure the levels of autophagic activity, we determined the expression of LC3B-II (as a model substrate for analysis of autophagy). LC3B-II protein expression increased 2 days and 4 days after 2-AA treatment, whereas NAC treatment reduced LC3B-II degradation (Fig. 9). Next, we monitored the degradation of sequestosome 1 (SQSTM)1/p62, which is an autophagic flux marker, because SQSTM1/p62 directly binds to LC3B and is selectively degraded by autophagy (42). We observed significant degradation of SQSTM1/p62 protein 4 days after 2-AA treatment, while NAC treatment restored the SQSTM1/p62 protein expression (Fig. 9). Our data suggest that 2-AA may promote atrophy via induction of the protein degradation pathway and autophagy in skeletal muscle.

### Inhibition of AMPK and proteosomal degradation in 2-AA-treated murine C2C12 myotubes reinstated muscle proteins to homeostatic levels.

To further elucidate the molecular pathway by which 2-AA promoted protein degradation and autophagy in skeletal muscle, we examined the effects of AMPK and proteosomal inhibitors as well as of NAC in the expression of the protein degradation pathway and muscle proteins in 2-AA-treated C2C12 myotubes. First, we confirmed the effect of 2-AA on the expression of AMPK, ubiquitin ligases, autophagy markers (ATG5, LC3B, and SQSTM1), MYH, and tropomyosin on the differentiated C2C12 myotubes. 2-AA significantly increased the expression of pAMPKβ1 (Fig. S4) and of MuRF1 and MAFbx (Fig. S5) between 24 and 48 h. Expression of ATG5 and LC3B protein was also significantly induced whereas SQSTM1/p62 expression was significantly downregulated between 12 and 24 h (Fig. S6). Degradation of MYH and tropomyosin was observed in 2-AA-treated C2C12 myotubes between 12 and 48 h (Fig. S7). These results further confirm and indicate the involvement of AMPK, ubiquitin ligases, and autophagy in the 2-AA-mediated skeletal muscle protein degradation that we observed *in vivo* in the present study.

10.1128/mBio.02211-19.4 FIG S4 2-AA treatment activates phosphorylation of AMPK in C2C12 myotubes. Immunoblotting of p-AMPKβ1 (Ser182) in C2C12 myotubes after 2-AA treatment was performed. β-Actin was used as the loading control. Histograms show the relative expression levels of proteins, and data are representative of results from three independent experiments. *n* = 3; values represent means ± SD; *P < *0.05, Student’s *t* test. Download FIG S4, TIF file, 2.2 MB. Copyright © 2019 Bandyopadhaya et al. 2019 Bandyopadhaya et al. This content is distributed under the terms of the Creative Commons Attribution 4.0 International license.

10.1128/mBio.02211-19.5 FIG S5 2-AA treatment modulates the ubiquitin-mediated proteosomal pathway in C2C12 myotubes. Immunoblotting of MuRF1 and MAFbx in C2C12 myotubes after 2-AA treatment was performed. β-Actin was used as the loading control. Histograms show the relative expression levels of proteins, and data are representative of results from three independent experiments. *n* = 3; values represent means ± SD; *P < *0.05, Student’s *t* test. Download FIG S5, TIF file, 2.0 MB. Copyright © 2019 Bandyopadhaya et al. 2019 Bandyopadhaya et al. This content is distributed under the terms of the Creative Commons Attribution 4.0 International license.

10.1128/mBio.02211-19.6 FIG S6 2-AA treatment induces autophagy in C2C12 myotubes. Immunoblotting of ATG5, LC3B (isoforms I and II), and SQSTM1/p62 in C2C12 myotubes after 2-AA treatment was performed. β-Actin was used as the loading control. Histograms show the relative expression levels of proteins, and data are representative of results from three independent experiments. *n* = 3; values represent means ± SD; *P < *0.05, Student’s *t* test. Download FIG S6, TIF file, 2.1 MB. Copyright © 2019 Bandyopadhaya et al. 2019 Bandyopadhaya et al. This content is distributed under the terms of the Creative Commons Attribution 4.0 International license.

10.1128/mBio.02211-19.7 FIG S7 2-AA treatment modulates muscle proteins in C2C12 myotubes. Immunoblotting of MYH and tropomyosin in C2C12 myotubes after 2-AA treatment. β-Actin was used as the loading control. Histograms show the relative expression levels of proteins, and data are representative of results from three independent experiments. *n* = 3; values represent means ± SD; *P < *0.05, Student’s *t* test. Download FIG S7, TIF file, 2.0 MB. Copyright © 2019 Bandyopadhaya et al. 2019 Bandyopadhaya et al. This content is distributed under the terms of the Creative Commons Attribution 4.0 International license.

**FIG 10:**
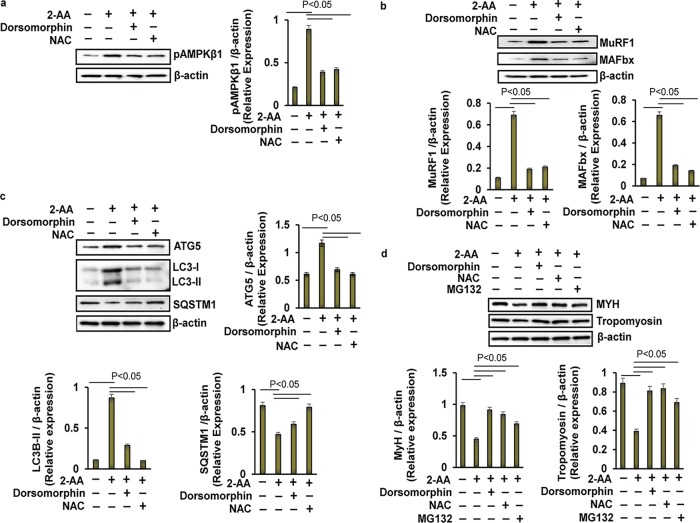
Effect of AMPK and proteasomal inhibitors on the 2-AA-mediated skeletal muscle degradation *in vitro*. Representative immunoblots of (a) pAMPKβ1, (b) ubiquitin ligases (MuRF1 and MAFbx), (c) autophagy markers (ATG5, LC3B, and SQSTM1), and (d) MYH and tropomyosin in 2-AA (400 μM)-treated mouse C2C12 myotubes with or without dorsomorphin (10 μM), with or without NAC (5 mM), and/or with or without MG132 (10 μM) for 24 h are shown. β-actin (bottom blot) was used as the loading control. Histograms show the relative expression levels of proteins, and data are representative of results from three independent experiments. *n* = 3, means ± SD. *P* < 0.05, one-way ANOVA.

Second, to investigate the role of AMPK in 2-AA-mediated skeletal muscle protein degradation, pAMPKβ1 levels were assessed in the presence of dorsomorphin, a selective inhibitor of AMPK (43). Panels a and b of Fig. 10 show that the 2-AA-mediated protein levels of AMPKβ1, MuRF1, and MAFbx (Fig. 10b) in the mouse myotubes were significantly reduced by dorsomorphin, reaching levels similar to their baseline expression levels. Similarly, addition of dorsomorphin reinstated the 2-AA-mediated increase of ATG5 and LC3B protein levels, reaching levels close to those observed at baseline, while it also resulted in partial recovery of SQSTM1/p62 expression in 2-AA-treated C2C12 myotubes (Fig. 10c). Moreover, dorsomorphin restored expression of both MYH and tropomyosin in 2-AA-treated C2C12 myotube cells (Fig. 10d). Thus, inhibition of 2-AA-mediated AMPK activation may lead to reduced proteasomal degradation and autophagy, thereby minimizing the skeletal muscle degradation in myotubes promoted by 2-AA.

To further elucidate the impact of 2-AA-mediated ROS on muscle protein degradation mediated via AMPK, phosphorylation of AMPKβ1 was investigated in the presence of NAC in 2-AA-treated C2C12 myotubes. NAC treatment dampened the 2-AA-mediated activation of AMPKβ1 (Fig. 10a) and restored expression of ubiquitin ligases (MuRF1 and MAFbx (Fig. 10b), as well as ATG5, LC3B, and SQSTM1/p62 (Fig. 10c), to almost baseline levels in 2-AA-treated myotubes. As expected, NAC treatment also restored the decreased MYH and tropomyosin protein expression in 2-AA-treated C2C12 mytobes. In corroboration, proteasomal inhibitor MG132 restored the MYH levels and, to a lesser extent, the tropomyosin levels in 2-AA-treated myotubes (Fig. 10d). These findings are in agreement with our *in vivo* findings and further support the notion that 2-AA-generated ROS triggers proteasomal degradation and skeletal muscle loss via regulation of AMPK activation and that this effect can be alleviated via NAC.

## DISCUSSION

**FIG 11:**
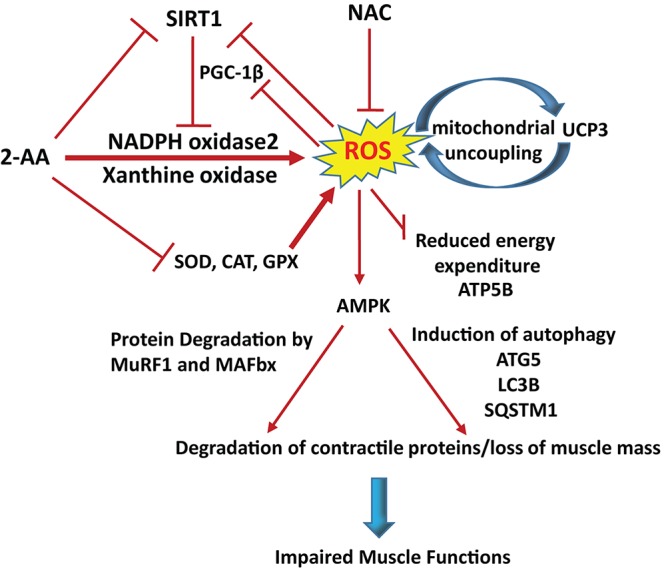
Proposed model of 2-AA’s action in skeletal muscle. 2-AA triggers ROS generation via activating XO activity and NOX2 expression and by impairing the activity of antioxidant enzymes as well as by downregulation of SIRT1 protein. Reduction of PGC-1β protein level impairs ROS detoxification, whereas upregulation of UCP3 gene (25) and protein levels impacts the mitochondrial functions (26), resulting in energy depletion. Energetic exhaustion and oxidative stress increases lead to muscle atrophy by activating AMPK, which controls autophagy and proteasomal degradation as assessed by the activation of autophagy markers ATG5, LC3B, and SQSTM1 and autophagy markers MuRF1 and MAFbx, respectively. NAC can ameliorate 2-AA-induced oxidative stress and thus rescue skeletal muscle protein degradation by scavenging the ROS.

Our results uncover further aspects of the biochemical and molecular processes that are potentially mediated by a bacterial QS molecule. A series of findings reported here support the notion of an effect of 2-AA on skeletal muscle atrophy via ROS overproduction and associated skeletal muscle dysregulation (Fig. 11). More specifically, this diagnostically important bacterial QS interkingdom infochemical promotes accumulation of ROS via dysregulation of the antioxidant defense system. Our data show that 2-AA induces ROS production by enhancing XO activity and NOX2 protein expression and dampens the enzymatic activity of SOD, catalase, and GPX, as well as the level of SIRT1 protein, resulting in increased oxidative stress. The 2-AA-mediated ROS accumulation may promote autophagy and protein degradation via activation of AMPK. These findings strongly support the muscle protein loss and atrophy model depicted in Fig. 11.

Perhaps the major finding of the present study was that the 2-AA-mediated reinforcement of the XO enzymatic activity and NOX2 protein expression in skeletal muscle might represent the initial source of ROS generation (Fig. 11). XO and NOX are known to be the nonmitochondrial sources of ROS in skeletal muscles (44). Previous *in vivo* studies have demonstrated that inhibition of XO activity diminishes the release of ROS and subsequently reduces muscle fatigue (45, 46). Recent studies have shown that suppression of XO activity can prevent muscular protein loss by inhibiting the atrogin-1 pathway and may induce resistance against muscular protein loss and atrophy in patients with sarcopenia or cachexia (47, 48). ROS generated from NOX has also been implicated in progressive skeletal muscle damage (12, 49).

The 2-AA-dependent downregulation of SIRT1 and PGC-1 is likely responsible for the induction of NOX2, which leads to the observed oxidative stress in the murine skeletal muscle. SIRT1 suppresses NOX-mediated oxidative stress through PGC-1 activation, which is sufficient to downregulate NOX2 expression (50). SIRT1 plays a crucial role against oxidative stress, including deacetylation of PGC1, which enhances detoxification, thus attenuating ROS (51). Several studies have demonstrated that decreased PGC-1 protein levels are associated with increased ROS generation and oxidative stress (52, 53). Interestingly, oxidative stress decreases expression of SIRT1 and its degradation (54, 55). The exact roles of SIRT1 and PGC-1 in 2-AA-mediated ROS accumulation need to be determined.

PGC-1α and PGC-1β have been shown to stimulate the gene expression of the mitochondrial proteins UCP2 and UCP3 (56), which have been shown to protect against ROS, dissipate the proton gradient, and attenuate mitochondrial superoxide formation potential. On the other hand, nonmitochondrial generation of ROS or ROS by-products can also activate UCP3 (57). Our data show that the levels of UCP3, a mostly skeletal muscle-specific protein, are increased despite the reduction in PGC-1β observed in skeletal muscle, suggesting that its activation primarily results from nonmitochondrial ROS generation.

Our findings corroborate our previous studies, as we found that 2-AA treatment downregulated electron transport system components and reduced the ATP synthesis rate in skeletal muscles (25). Depletion of SIRT1 suppresses cellular ATP levels (58). The level of ATP generated by mitochondria was reduced with higher concentrations of ROS and oxidative stress (49, 59). Interestingly, it was previously shown that ROS downregulates the H^+^-ATP synthase function (60), and we found that depletion of ROS with NAC in 2-AA-treated mice resulted in a partial rescue of ATP5b expression in skeletal muscle. These findings further emphasize the impact of 2-AA on the tightly regulated connections that exist among ATP synthase, energy production, ROS generation for mitochondrial function, and skeletal muscle physiology and homeostasis.

Our previous and current findings robustly indicate that 2-AA induces mitochondrial dysfunction and oxidative stress in skeletal muscle, presumably due to suppression of the antioxidant defense and detoxification circuits, resulting in accumulation of ROS (25, 26). Although more studies are needed to determine the direct target of this molecule in ROS generation, our data strongly suggest that the initial source of ROS accumulation is nonmitochondrial and involves XO and NOX2.

Pulmonary P. aeruginosa infection in CF patients promotes skeletal muscle atrophy via proteasomal proteolytic pathways and autophagy (17, 61, 62). Recent studies demonstrated that localized accumulation of ROS can activate autophagic pathway and cause muscle atrophy (63). Our studies indicated that downregulation of PGC-1β, which is reported to preferentially induce genes involved in the removal of ROS, may lead to myotube atrophy as a result of the promoted oxidant stress. Reduced ATP and increased oxidative stress levels have been reported to activate AMPK (37, 38), leading to myotube atrophy through the activation of proteasomal degradation pathway involving MuRF1 and MAFbX, which are also activated by oxidative stress (64, 65). Consistently, our results show that not only autophagy markers ATG5, LC3B, and SQSTM1 but also ubiquitin ligases MuRF1 and MAFbx of the protein degradation pathway are activated following 2-AA treatment, suggesting that these changes may promote myotube atrophy through the activation of proteasomal degradation and autophagy.

### Conclusion.

The data presented here suggest a novel mode of action and mechanistic insights into the P. aeruginosa QS-regulated infochemical 2-AA, which promotes ROS accumulation and skeletal muscle protein dysregulation, leading to impaired skeletal muscle function. Our findings suggest that the following actions could be taken to avoid such an impairment: (i) inhibition of the activity of XO could reduce the initial induction of nonmitochondrial ROS; (ii) restoration of the function of SIRT1 and PGC-1β could rescue mitochondrial function and inhibition of NOX2; (iii) antioxidant treatment could further reduce the muscle atrophy; and (iv) as muscle proteins are impacted by the 2-AA-mediated inhibition of ubiquitin ligases, maintenance of these important sarcomeric proteins could prove to be beneficial in the context of infection. The oxidative stress and muscle protein loss promoted by 2-AA may be highly relevant in the clinical setting and, more specifically, in patients with acute and chronic infections, as well as those with CF, who are prone to chronic and repeated infection episodes. The fact that QS is highly conserved across prokaryotes makes it possible that 2-AA-like molecules with similar effects exist in other pathogens as well and raises interesting questions about the potential role of small QS molecules in the chronic fatigue syndrome.

## MATERIALS AND METHODS

### Ethics statement.

The animal protocol used was approved by Institutional Animal-Care and Use Committee (IACUC) of Massachusetts General Hospital (approval no. 2006N000093). No randomization or exclusion of data was performed. All methods were performed in accordance with the relevant guidelines and regulations.

### Experimental animals.

Six-week-old male CD1 mice (Charles River Laboratory) were injected intraperitoneally (i.p.) with 2-AA (Sigma-Aldrich, USA) (6.75 mg/kg of body weight) once. N-Acetyl-l-cysteine (NAC) (Sigma-Aldrich) (10 mg/kg; i.p.) was given once per day for 3 days (Fig. 1a). For sham control animals, phosphate-buffered saline (PBS) was injected i.p. The mouse was maintained with free access to food and water during the whole experimental period. The mouse gastrocnemius muscle was collected and harvested for molecular and biochemical studies.

### Cell culture and treatments.

The murine skeletal muscle cell line, i.e., C2C12 myoblasts from American Type Culture Collection, was maintained in high-glucose Dulbecco’s modified Eagle’s medium (DMEM; Thermo Fisher Scientific, USA) supplemented with 10% fetal bovine serum (Thermo Fisher Scientific) containing 100 U/ml penicillin and 100 μg/ml streptomycin in the presence of 5% CO_2_ at 37°C. The cells were seeded in T-75 tissue culture flasks (BD Falcon, USA) and used between passages 2 and 3. After reaching approximately 70% confluence, the cells were switched to differentiation medium composed of high-glucose DMEM with 2% horse serum (Thermo Fisher Scientific) in order to induce myoblast fusion into differentiated myotubes for 48 h. The myotubule formation was monitored by the use of a phase-contrast microscope (see Fig. S1 in the supplemental material).

Differentiated C2C12 myotubes were treated with 2-AA (Enamine Ltd., Ukraine) (200, 400, and 800 μM) at different time points. For the inhibitor assay, cells were pretreated with allopurinol (Abcam, USA) (50 μM), apocynin (Abcam) (10 μM), dorsomorphin (Abcam) (10 μM), gp91ds-tat (Anaspec Inc.) (50 μM), MG132 (Sigma-Aldrich) (10 μM), or NAC (Sigma-Aldrich) (5 mM) for 1 h before 2-AA treatment.

Cell viability was assessed by the MTT [3-(4,5-dimethyl-2-thiazolyl)-2,5-diphenyl-2H-tetrazolium bromide]; Sigma-Aldrich) assay as previously described (30).

### Measurement of oxidative stress markers. (i) Total ROS.

For measurement of the ROS content in tissues, we used an OxiSelect *in vitro* ROS/RNS assay kit (Cell Biolabs Inc., San Diego, CA) according to the manufacturer’s protocol. In brief, the gastrocnemius muscle of the mice was homogenized in ice-cold PBS. Homogenates were centrifuged at 10,000 rpm for 5 min to obtain the supernatants for ROS content measurement. A 50-μl volume of supernatant was added to a well of a 96-well plate; then, 50 μl of catalyst was added to each well, the well contents were mixed thoroughly, and the reaction mixture was incubated for 5 min at room temperature. A 100-μl volume of fluorescent probe 2,7-dichlorofluorescin diacetate (DCFH-DA) was added to each well followed by incubation at room temperature under dark conditions for 30 min. The relative levels of fluorescence of the samples and the standards were measured using a Tecan plate reader (excitation wavelength of 484 nm; emission wavelength of 530 nm). ROS production was expressed as arbitrary fluorescence units (AFU).

**(ii) Cellular ROS levels.** ROS levels in myotubes were measured using the Cellular ROS/Superoxide Detection assay kit (catalog no. ab139476; Abcam). The C2C12 myotube culture conditions and treatments were the same as described above. Following treatment, the cells were labeled with fluorescent reporter dyes, which are oxidized by ROS with high specificity, according to the manufacturer’s instructions. For total ROS detection we used the cell permeant reagent DCFH-DA, a fluorogenic dye that measures total ROS activity within the cell. Pyocyanin was used as positive control (Fig. S2). After incubating at 37°C for 20 min, absorbance was measured at 450 nm.

**(iii) Total antioxidant capacity (TAC).** TAC was measured in samples of frozen muscle tissue using a TAC assay kit (catalog no. ab65329; Abcam, USA) according to the manufacturer’s protocol. Briefly, tissues were suspended in ice-cold PBS and homogenized and the homogenate was centrifuged at 14,000 rpm for 5 min at 4°C. A 100-μl volume of Cu^2+^ working solution was added to the homogenate and the standards in the microtiter plate. The plate was kept at room temperature for 90 min. The colorimetric probe chelates Cu^+^ ion and produces an absorbance peak at 570 nm. The samples were assayed in conjunction with a series of standards, and TAC was determined by interpolation of the standard curve.

### Measurement of antioxidant system. (i) SOD activity.

SOD activity was assessed in gastrocnemius muscle tissues by the use of a colorimetric assay kit (catalog no. ab65354; Abcam) as described in the manufacturer’s protocol. The tissue was perfused in PBS and homogenized in ice-cold 0.1 M Tris-HCl (pH 7.4) (containing 0.5% Triton X-100, 5 mM β-mercaptoethanol, 0.1 mg/ml phenylmethylsulfonyl fluoride). The homogenate was centrifuged at 14,000 × *g* for 5 min at 4°C, and the supernatant was collected. The supernatant, controls, and standards were added to separate wells of microtiter assay plates; then, 200 μl of water-soluble tetrazolium salt (WST) working solution was added to all wells and 20 μl of enzyme working solution was added to the wells containing supernatants and to the proper controls only, according to the manufacturer’s instructions. The plates were then incubated at 37°C for 20 min before being read on a microplate reader (Tecan) at 450 nm. WST-1 reduction is inhibited by SOD, which catalyzes the dismutation of the superoxide radicals to generate H_2_O_2_ and O_2_. Therefore, SOD activity was calculated on the basis of percent inhibition of WST-1 reduction, which in turn reflected percent inhibition of the superoxide anions.

**(ii) Catalase activity.** Catalase activity in the gastrocnemius muscle tissues was measured with the catalase assay kit (catalog no. ab83464; Abcam) according to the manufacturer’s protocol. The tissue was homogenized in 200 μl cold assay buffer and centrifuged for 10 min at 14,000 rpm. Clear supernatant was used for the assay as described in the protocol. After 30 min incubation time the reaction was stopped, and the optical density was measured with a Tecan at 570 nm. The catalase enzyme activity was expressed in nanomoles per milligram of protein.

**(iii) GPX activity.** Muscle tissue was homogenized in 50 mM Tris-HCl buffer (pH 7.5) (containing 5 mM EDTA and 1 mM dithiothreitol). The homogenate was then centrifuged at 10,000 × *g* for 15 min at 4°C. GPX activity was analyzed calorimetrically in 20 μl of supernatant per well in a microtiter plate using a glutathione peroxidase assay kit (catalog no. 703102; Cayman Chemical, USA) according to the manufacturer’s instructions. The assay evaluates GPX activity indirectly by the use of a reaction coupled with glutathione reductase, which generates NADP^+^. This process is accompanied by a decrease in absorbance measured at 340 nm in kinetic mode. The absorbance decrease rate is directly proportional to the GPX activity of each sample, and the result is expressed in nanomoles per minute per milligram of protein.

**(iv) GST activity.** GST activity in the tissues was measured using a colorimetric GST activity assay kit (catalog no. ab65326; Abcam) according to the manufacturer’s protocol. The tissue was homogenized in GST assay buffer, and the homogenate was centrifuged (at 10,000 × *g* for 15 min at 4°C). A 50-μl volume of homogenate was mixed with 50 μl of GST substrate CDNB (1-chloro-2,4-dinitrobenzene) mix. GST-catalyzed reactions between GSH and the GST substrate produce a dinitrophenyl thioester which can be detected spectrophotometrically in a Tecan reader at 340 nm.

### Assessment of XO activity.

An assay kit (catalog no. ab102522; Abcam) was used to monitor XO activity according to the manufacturer’s instructions. Briefly, tissues were homogenized in 100 mM Tris-HCl buffer (pH 7.5) and centrifuged (10,000 × *g* for 15 min in 4°C) to get the supernatants. The working solutions were added to 50-μl volumes of supernatants and were incubated at 37°C for 30 min. The fluorescence measurement was performed in a microplate reader (Tecan) at 570 nm at intervals of 10 min for 1 h. For the standard, XO was used as supplied in the kit, and XO activity was determined by comparing the fluorescence of the supernatants with that of the standards. The specific activity was expressed as nanomoles per minute per milligram of protein.

### Western blotting.

Tissue extracts were prepared in radioimmunoprecipitation assay (RIPA) buffer (Cell Signaling Technology, USA). The resulting preparations (10 to 20 μg) were then fractionated by polyacrylamide-SDS gel electrophoresis and immunoblotted with SIRT-1 (sc-15404), PGC-1β (sc-373771), ATP5B (sc-55597), MuRF1 (sc-32920), MAFbx (sc-33782), tropomyosin (sc-28543), MYH (sc-20641), hypoxanthine phosphoribosyltransferase (HPRT) (sc-20975), NOX2 (gp91-phox) (sc-130543), and β-actin (47778) from Santa Cruz Biotechnology, Inc., USA; with UCP-3 (catalog no. 97000), pAMPKβ1 (Ser182) (catalog no. 4186), and AMPKβ1 (catalog no. 4150) from Cell Signaling Technology, USA; with ATG5 (NBP2-24389) and LC3B (NB100-2220) from Novus Biologicals, USA; and with anti-SQSTM1/p62 (catalog no. ab56416) from Abcam, USA. The primary and secondary antibodies were used at dilutions of 1:1,000 and 1:10,000, respectively. The blots were visualized with SuperSignal West Pico chemiluminescent substrate (Thermo Scientific, Rockford, IL, USA), according to the manufacturer’s instructions. The immunoreactive bands were analyzed using ImageJ software, and a densitometry analysis was conducted.

### Protein assay.

A bicinchoninic acid (BCA) protein assay kit (Thermo Fisher Scientific, USA) was used to estimate the protein concentrations in the resultant supernatants per the manufacturer’s instructions.

### Statistical analysis.

Wherever applicable, at least three independent experiments were performed, and the data were analyzed using one-way analysis of variance (ANOVA) with Tukey’s honestly significant difference (HSD) *post hoc* test, as appropriate. For all experiments, *P* values of <0.05 were considered significant.
