# Erratum for Bandyopadhaya et al., “Pseudomonas aeruginosa Quorum Sensing Molecule Alters Skeletal Muscle Protein Homeostasis by Perturbing the Antioxidant Defense System”

**DOI:** 10.1128/mBio.03316-19

**Published:** 2020-02-04

**Authors:** Arunava Bandyopadhaya, A. Aria Tzika, Laurence G. Rahme

**Affiliations:** a Department of Surgery, Center for Surgery, Innovation and Bioengineering, Massachusetts General Hospital, Harvard Medical School Boston, Boston, Massachusetts, USA; b Shriners Hospitals for Children Boston, Boston, Massachusetts, USA; c Athinoula A. Martinos Center of Biomedical Imaging, Massachusetts General Hospital, Boston, Massachusetts, USA; d Department of Microbiology, Harvard Medical School, Boston, Massachusetts, USA

## ERRATUM

Volume 10, no. 5, e02211-19, 2019, https://doi.org/10.1128/mBio.02211-19. The second paragraph under Acknowledgments (PDF page 15) should have read as follows: L.G.R. has a financial interest in Spero Therapeutics, a company developing therapies for the treatment of bacterial infections. L.G.R.’s financial interests were reviewed and are managed by Massachusetts General Hospital and Partners HealthCare in accordance with their conflict of interest policies. The rest of us declare that we have no competing interests.

