# Supplementary figures and images for: Pseudomonas aeruginosa Quorum Sensing Molecule Alters Skeletal Muscle Protein Homeostasis by Perturbing the Antioxidant Defense System
Source: mBio. 2019 Oct 1;10(5):e02211-19. doi: 10.1128/mBio.02211-19 (PMC6775459; doi:10.1128/mBio.02211-19)

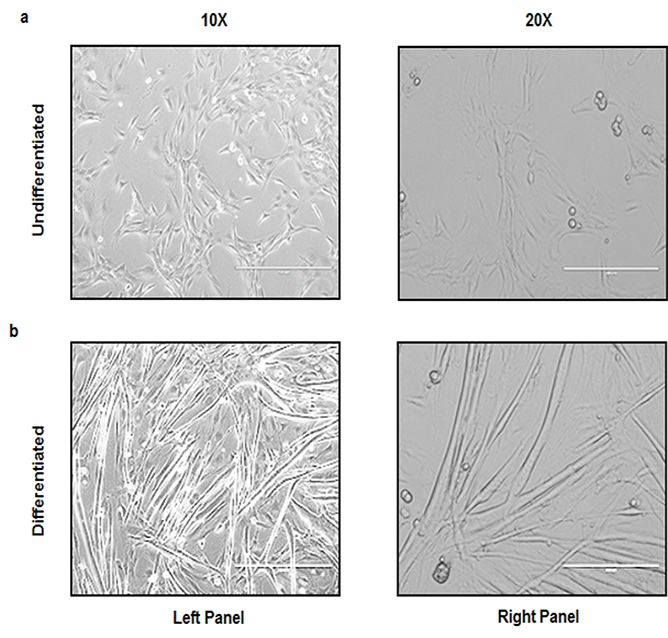

Supplement: FIG S1 [file mBio.02211-19-sf001.tif]

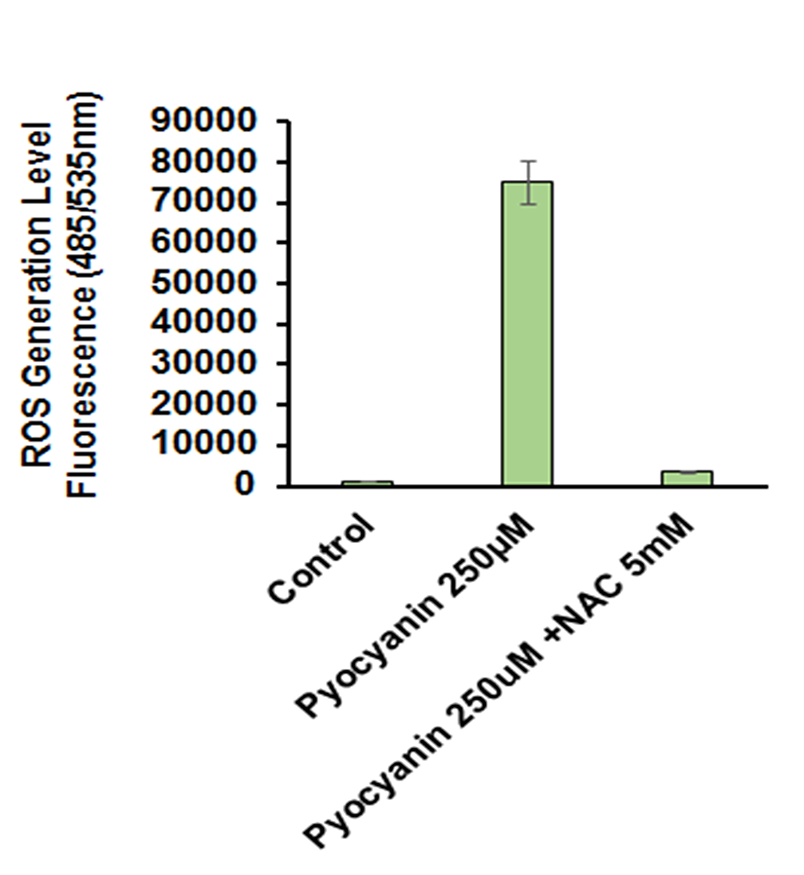

Supplement: FIG S2 [file mBio.02211-19-sf002.tif]

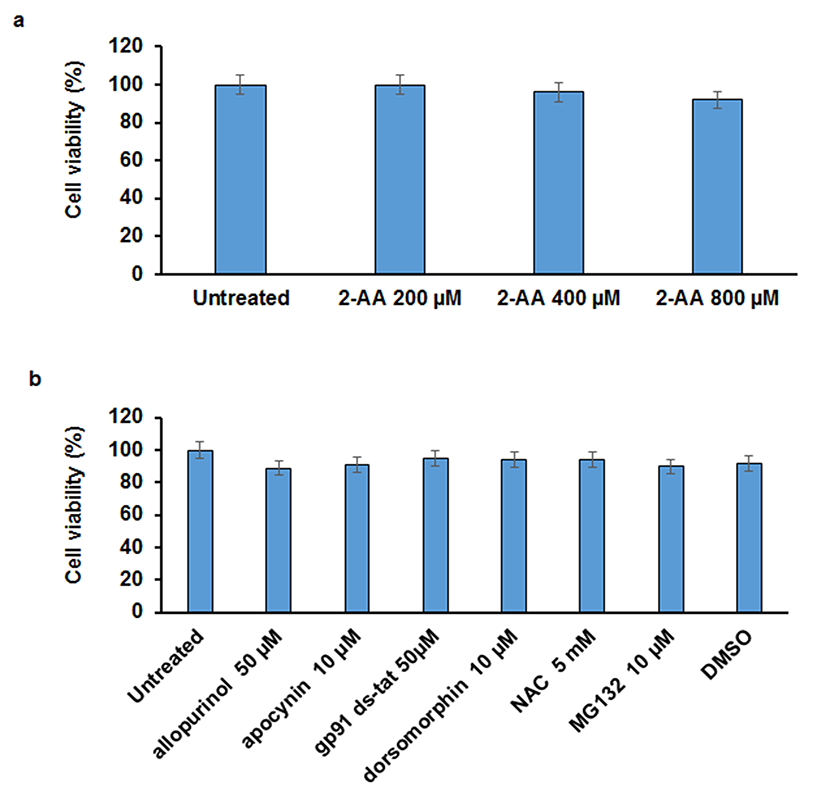

Supplement: FIG S3 [file mBio.02211-19-sf003.tif]

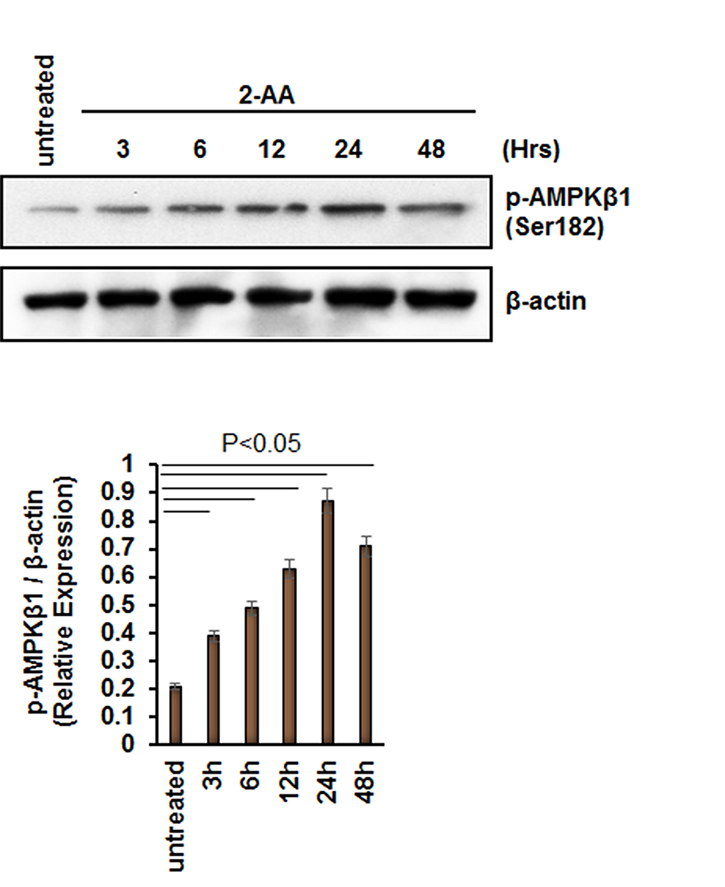

Supplement: FIG S4 [file mBio.02211-19-sf004.tif]

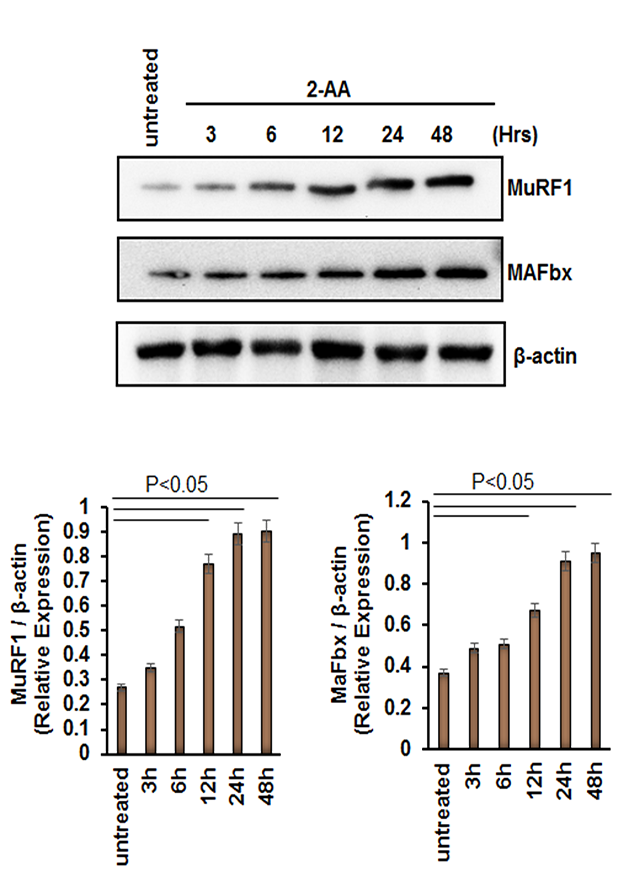

Supplement: FIG S5 [file mBio.02211-19-sf005.tif]

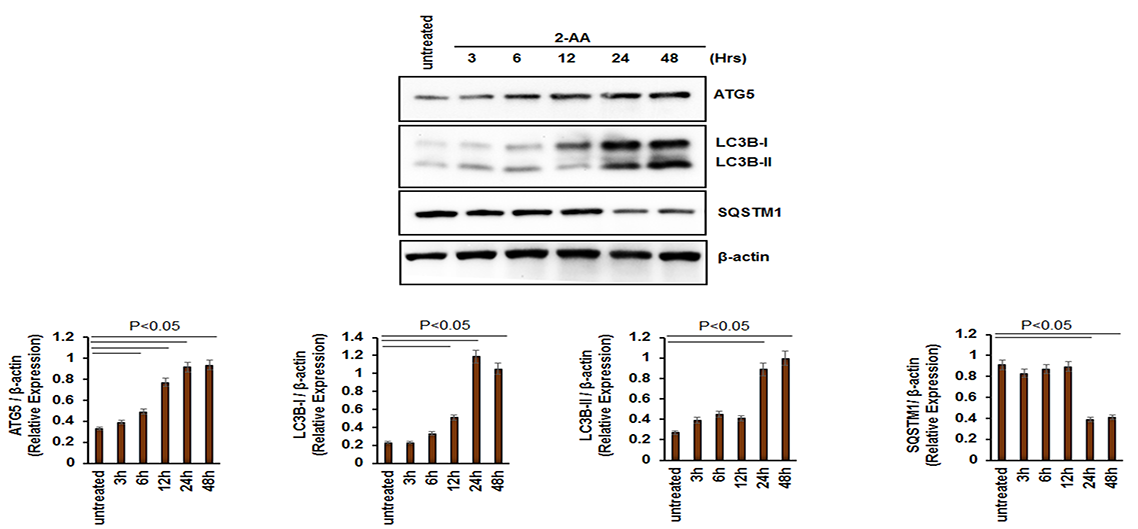

Supplement: FIG S6 [file mBio.02211-19-sf006.tif]

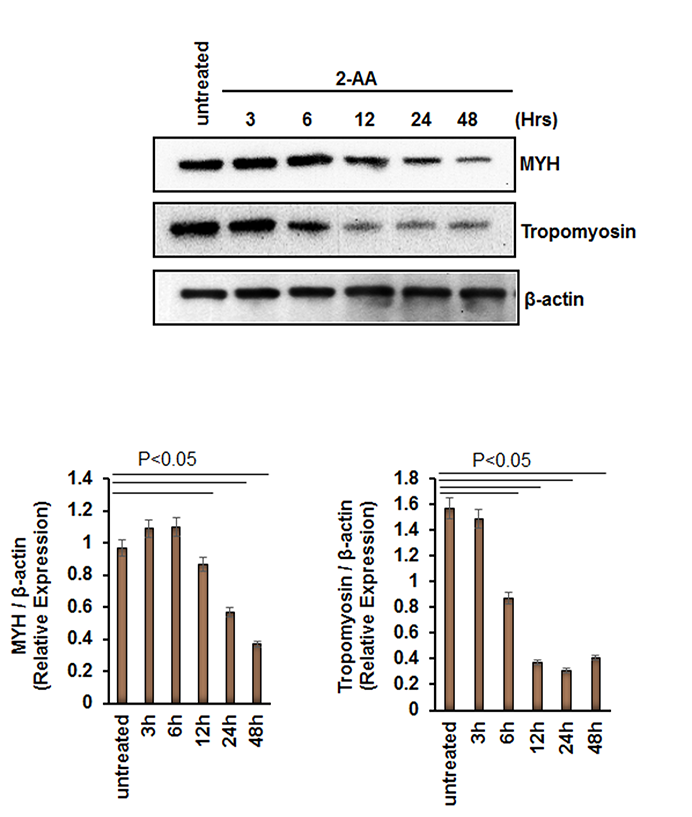

Supplement: FIG S7 [file mBio.02211-19-sf007.tif]
